# Modification in the Composition of *Lactuca sativa* L. Plants Exposed to Abiotic Stress Induced by Commonly Used Antibiotics

**DOI:** 10.3390/plants14060842

**Published:** 2025-03-07

**Authors:** Ildiko Lung, Maria-Loredana Soran, Aura Nicoleta Sârb, Adina Stegarescu, Augustin C. Moț, Iolanda-Veronica Ganea, Delia-Maria Gligor, Ocsana Opriș

**Affiliations:** 1 National Institute for Research and Development of Isotopic and Molecular Technologies, 67-103 Donat, 400293 Cluj-Napoca, Romania; ildiko.lung@itim-cj.ro (I.L.); loredana.soran@itim-cj.ro (M.-L.S.); adina.stegarescu@itim-cj.ro (A.S.); iolanda.ganea@itim-cj.ro (I.-V.G.); 2 Faculty of Environmental Sciences and Engineering, Babeș-Bolyai University, 30 Fântânele, 400000 Cluj-Napoca, Romania; sirbaura@gmail.com (A.N.S.); delia.gligor@ubbcluj.ro (D.-M.G.); 3 Department of Chemistry, Babes-Bolyai University, 400028 Cluj-Napoca, Romania; augustin.mot@ubbcluj.ro

**Keywords:** abiotic stress, assimilatory pigments, antibiotics, antioxidant capacity, bioactive compounds, elemental content, lettuce, polyphenols

## Abstract

The widespread worldwide use of antibiotics leads to significant diffuse pollution of the environment, but the understanding of the effects of many important antibiotics on plants is still limited. This study aimed to gain insight into the impact of ampicillin (AMP) and ciprofloxacin (CIP) on the bioactive compounds, antioxidant capacity, and elemental content of lettuce (*Lactuca sativa* L.). The lettuce was grown in a climate chamber under controlled conditions of light and temperature, in soil with contaminated antibiotics of different concentrations (7.5 mg kg^−1^—AMP I; 15 mg kg^−1^—AMP II; 30 mg kg^−1^—AMP III; 5 mg kg^−1^—CIP I; 10 mg kg^−1^—CIP II; and 20 mg kg^−1^—CIP III). The results obtained for the plants grown in the presence of antibiotics were compared with the control plants. Changing the growth conditions influenced the composition of the biologically active compounds and the content of elements selected in this study. Thus, it was observed that the plants grown with antibiotics exhibited a double increase in polyphenolic content, especially at higher treatment levels, but also a significant decrease in assimilatory pigments (up to 70.19%), 2,2-Diphenyl-1-picrylhydrazyl (DPPH) radical-bleaching activity (up to 83.80%), and elemental contents compared to the control plants. Multivariate analysis was used to gain insight into similarities and differences between treatments and the association of the tested variables following the applied treatments, indicating a coping mechanism of the plants against the antibiotic treatments.

## 1. Introduction

Antibiotics represent a real advance in medicine, and their discovery has led to the modification of old therapeutic concepts. They are widely used in human medicine, veterinary medicine, and aquaculture to prevent or treat microbial infections. The most frequently used antibiotics are β-lactams, sulfonamides, tetracyclines, and macrolides [1]. Their overuse and wrong application have led to contamination with antibiotics, with large quantities of them being present in manure, soils, wastewater, and water basins [2].

Antibiotics can be absorbed by plants from the soil and have a significant impact on human health. Also, several studies have shown that antibiotics can harm animals and plants that ingest them through contaminated water. However, understanding of the effects on plants is limited.

Lettuce (*Lactuca sativa* L.), belonging to the *Asteraceae* family, was chosen for this study because it is a well-known leafy vegetable, widespread worldwide for its use in salad preparation, and presents sensitivity to environmental contaminants [3]. The chemical composition of the plant revealed the presence of different classes of secondary metabolites, such as phenols, terpenoids, and flavonoids, which should be responsible for its biological activities. In addition, the plant contains essential elements such as minerals, as well as vitamins and organic substances [4].

In the literature, there are only a few studies on the effect of antibiotics on lettuce. Danilova et al. [2] found that oxytetracycline reduced chlorophylls, biomass, and the root and stem length of lettuce grown in soil and hydroponics spiked with different concentrations of antibiotics. The capacity of lettuce (*Lactuca sativa* L.) and carrot (*Daucus corota* L.) to absorb tetracycline and amoxicillin from irrigated water was investigated by Azanu et al. [5]. The study was carried out in pots that were irrigated with known concentrations of antibiotics. The tested antibiotics were detected in the two studied plants, being detected in a higher concentration in the carrot. Three different concentrations of tetracycline, oxytetracycline, chlortetracycline, sulfamethazine, sulfamethoxazole, and sulfadimethoxine were used to determine their effect on cucumber (*Cucumis sativus* L.), cherry tomato (*Solanum lycopersicum* L.), and lettuce (*Lactuca sativa* L.). The experiment was conducted in a greenhouse for 45 days, with the plants being grown in pots filled with sandy clay soil. Following the experiment, it was found that all antibiotics had negative effects on plant growth [6]. In a study conducted by Choe et al. [7], lettuce seedlings were exposed to various doses of chlortetracycline for 4 weeks in a hydroponic system. They investigated the defense response of lettuce to chlortetracycline toxicity by exploring several antioxidant regulatory metabolites, hormones, enzymes, and genes. Wahid et al. [8] investigated the effects of ampicillin and ofloxacin on *Lactuca sativa* L. germination and growth when the plant was grown in soils treated with three organic amendments (compost, rice husk, and vermicompost). The results indicated that by adding compost, the root length of the ampicillin-exposed plant increased significantly, while by adding vermicompost, the shoot length of the ofloxacin-exposed seedlings increased by 64%.

Among the most widely used antibiotics are fluoroquinolones and β-lactam antibiotics [8]. Ciprofloxacin (CIP), a synthetic fluoroquinolone antibiotic, is used globally for the management of bacterial infections in both humans and animals [9]. Ampicillin (AMP), a β-lactam antibiotic, is one of the most consumed antibiotics for the treatment of infections with Gram-negative and -positive bacteria [10]. The two antibiotics were chosen for this study because they are widely used.

To our knowledge, there are no studies regarding the effects of CIP and AMP on bioactive compounds, as well as on element contents (macro- and microelements) in lettuce. Therefore, this study aims to evaluate how bioactive compound content, antioxidant activity, and elemental composition in lettuce (*Lactuca sativa* L.) plants respond to soil contamination with different concentrations of antibiotics. Specifically, the study investigates the impact of antibiotic presence on chlorophyll and carotenoid content, polyphenols, and elemental content in the selected plants. We hypothesized that higher concentrations of antibiotics lead to a plant response to stressors. The research also aims to assess how these changes can affect lettuce plants and raise awareness of the potential consequences for food quality and ecosystem health in agricultural systems contaminated with antibiotics.

## 2. Results and Discussion

### 2.1. Modification in the Composition of Lactuca sativa L. Plants Exposed to Antibiotic Treatments

#### 2.1.1. Assimilatory Pigments

**Figure 1:**
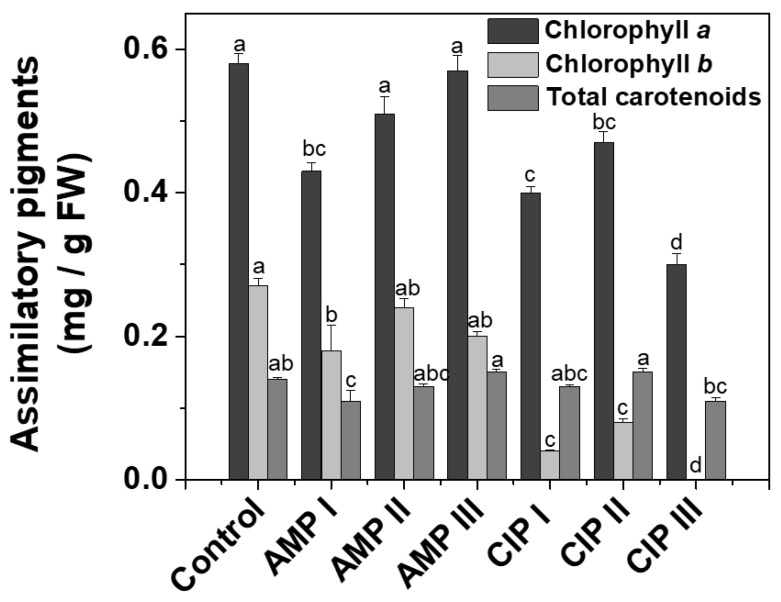
Comparative diagram of assimilatory pigments (chlorophyll *a*, chlorophyll *b*, and total carotenoids) determined in the *Lactuca sativa* L. plants (mg/g of fresh weight (FW) plant) exposed to treatments with ampicillin (AMP I—7.5 mg kg^−1^; AMP II—15 mg kg^−1^; AMP III—30 mg kg^−1^) and ciprofloxacin (CIP I—5 mg kg^−1^; CIP II—10 mg kg^−1^; CIP III—20 mg kg^−1^). The control is represented by the plants untreated with antibiotics. Every point represents the mean ± standard error of the mean obtained from three independent replicate experiments. The letters above the columns indicate grouping information using the Tukey method, and the means that do not share a letter are considered significantly different.

Chlorophylls are a key factor influencing the photosynthesis process, which in turn has a direct effect on plant growth and development [11]. A decrease in chlorophyll content is a common stress response that leads to reduced light absorption [12]. The results obtained for chlorophyll (*a* and *b*) and total carotenoids after the quantitative determination are presented in Figure 1. The content of chlorophylls (Figure 1) decreased in plants grown in soil contaminated with AMP and CIP, in comparison with control plants. Chlorophyll *a* decreased statistically significantly in the plants grown with AMP I (25.74%), CIP I (29.98%), CIP II (18.41%), and CIP III (47.24%), compared to control lettuce plants. The decreases were more important in the case of the chlorophyll *b* content. Chlorophyll *b* content was 33.26% (AMP I), 85.27% (CIP I), and 70.19% (CIP II) lower than in the control lettuce plants. For the plants treated with CIP III, chlorophyll *b* was not detected. These behaviors indicate a stress response of the plants to the antibiotic treatment. In their 2018 study, Singh et al. showed that applying amoxicillin inhibited the leaf growth of *Spirodela polyrhiza* L. and caused a significant decrease in the levels of photosynthetic pigments, proteins, starch, and lipids [13]. Moreover, the increase in the ratio between chlorophyll *a* and *b* indicates the presence of chemical stress in the plants [14].

Carotenoids in plants are important lipid-soluble antioxidants, but they also play a key role as integral components of pigment-binding complexes, light harvesting, and excess energy dissipation [15,16]. In our study, we found that total carotenoids significantly decreased only in plants treated with AMP I (26.73%). Under severe stress conditions, carotenoids can be rapidly degraded, leaving them unavailable to protect the plant from oxidative damage and photoinhibition [12].

#### 2.1.2. Total Polyphenols

**Figure 2:**
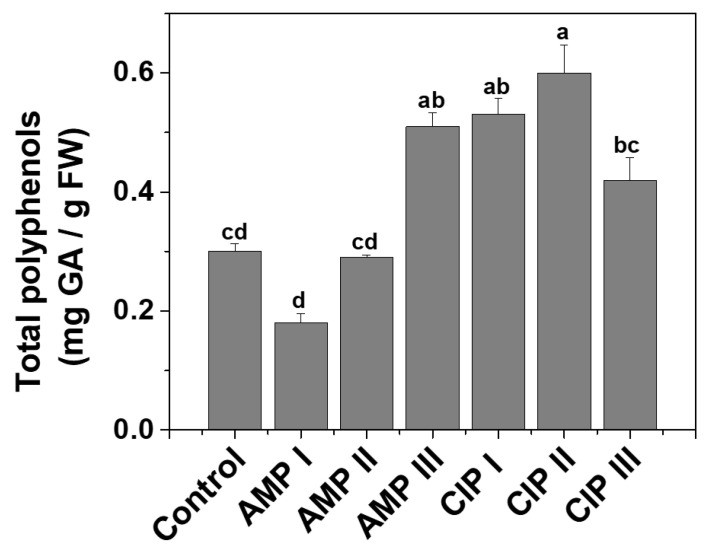
Total polyphenolic content, mg gallic acid (GA)/g fresh weight (FW), in the extracts of *Lactuca sativa* L. leaves grown in the presence of antibiotics (ampicillin—AMP; ciprofloxacin—CIP) of different concentrations (AMP I—7.5 mg kg^−1^; AMP II—15 mg kg^−1^; AMP III—30 mg kg^−1^) and ciprofloxacin (CIP I—5 mg kg^−1^; CIP II—10 mg kg^−1^; CIP III—20 mg kg^−1^). Every point represents the mean ± standard error of the mean obtained from three independent replicate experiments. The letters atop the columns stand for statistical differences, as in Figure 1.

A calibration curve was created using gallic acid (GA) as a standard solution to determine the total amount of polyphenols. The concentration range for GA was between 0.001 and 0.800 mg mL^−1^. The total polyphenolic content in lettuce leaves was calculated using the calibration curve equation y = 0.5046x + 0.0016 (R^2^ = 0.9993). The total polyphenolic compounds, shown in Figure 2, were expressed as mg of gallic acid (GA) per gram of fresh weight (FW) of *Lactuca sativa* L. leaves.

In comparison to the control, it was found that the total polyphenols decreased for the plants grown in soil contaminated with AMP I (38.53%) and approximately doubled in the case of plants grown in soil contaminated with AMP III, CIP I, CIP II, and CIP III. This behavior supports the hypothesis that in the case of AMP, although at low levels of treatment, the biosynthesis of polyphenols is inhibited, and at higher levels, the plants have to cope with stress via polyphenol upregulation. In the case of CIP, this is valid at all three applied levels.

#### 2.1.3. Antioxidant Capacity as DPPH Radical Bleaching

**Figure 3:**
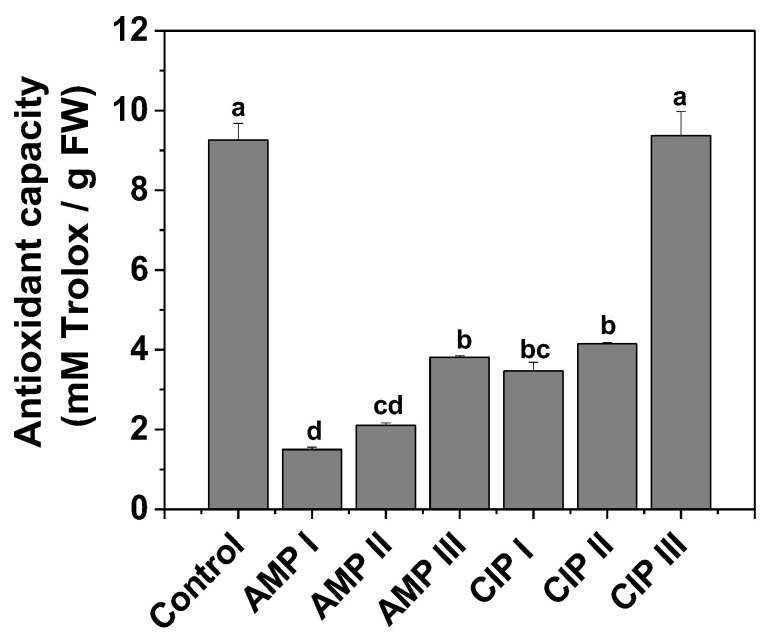
Antioxidant capacity, mM Trolox/g fresh weight (FW) of *Lactuca sativa* L. extracts. The lettuce leaves were treated with antibiotics (ampicillin—AMP; ciprofloxacin—CIP) of different concentrations (AMP I—7.5 mg kg^−1^; AMP II—15 mg kg^−1^; AMP III—30 mg kg^−1^); and ciprofloxacin (CIP I—5 mg kg^−1^; CIP II—10 mg kg^−1^; CIP III—20 mg kg^−1^). The control is represented by the antioxidant capacity determined in the lettuce plants untreated with antibiotics. Every point represents the mean ± standard error of the mean obtained from three independent replicate experiments. The letters atop the columns stand for statistical differences, as in Figure 1.

The antioxidant activity of the plant extracts was assessed using a calibration curve created with varied concentrations of Trolox (0–400 µM). Therefore, the calibration curve equation y = 0.1960x + 0.0044 (R^2^ = 0.9991) was applied to determine the antioxidant capacity of the polyphenolic lettuce extracts. The results for antioxidant capacity, presented in Figure 3, are expressed in mM Trolox equivalents (mM Trolox/g FW).

Antioxidant capacity significantly decreased in plants grown in soil contaminated with AMP or CIP compared to control plants. Although the polyphenol content increased in some samples, surprisingly, the radical-bleaching ability was drastically diminished. This could be explained by the change in the polyphenol profile, such as the diminishing in the radical-bleaching polyphenols and the increase in other types of polyphenols that are more beneficial for the plant to cope with applied chemical stress. The exception is the plants grown in soil contaminated with CIP III, in which the antioxidant capacity enhanced statistically insignificantly relative to the control plants.

### 2.2. Variation of the Bioactive Compounds and Antioxidant Capacity in Treated Lactuca sativa L. Plants Relative to Control

To determine the effect of the studied antibiotics on the bioactive compounds and antioxidant capacity of lettuce, the findings for the plants grown in contaminated soil were compared with the results obtained for the control plants. In this way, it was determined how the antibiotic, as well as its concentration added to the soil, influences the increase or decrease in the content of bioactive compounds and the antioxidant capacity (Table 1). It can be observed that in the examined plants, relative to the control, both increases and decreases were obtained in terms of the amount of bioactive compounds and antioxidant capacity. Thus, an increase in the content of polyphenols was recorded in the case of plants grown in soil contaminated with AMP III (0.68 times), CIP I (0.74 times), CIP II (0.97 times), and CIP III (0.37 times). An increase was also recorded in the case of plants grown in soil contaminated with AMP III (0.05 times) and CIP II (0.05 times) in terms of the content of total carotenoids, as well as in the case of plants grown in soil contaminated with CIP III (0.01 times), in terms of antioxidant capacity.

In all other cases, there was a decrease in the amount of bioactive compounds and antioxidant capacity. The biggest decreases were recorded in the case of plants grown in soil contaminated with CIP I (0.85 times), CIP II (0.70 times), and CIP III (0.98 times), in relation to the amount of chlorophyll *b* and in the case of plants developed in soil contaminated with AMP I (0.84 times), AMP II (0.77 times), AMP III (0.59 times), CIP I (0.63 times). and CIP II (0.55 times), for the antioxidant capacity.

### 2.3. Elemental Composition of Lactuca sativa L. Plants

The element content in plant tissues has been shown to correlate with the performance of individual plants and to impact the structure and function of entire ecosystems [1]. For proper development, plants need elements in both substantial amounts (macroelements: N, P, S, K, Mg, and Ca) and trace amounts (microelements: Fe, Zn, Mo, Cu, Mn, Cr, Ni, and B). When absorbed in the right quantities, these elements are essential components of proteins, plant cell walls, and chlorophylls in plants. They also play an important role in different processes, such as photosynthesis, osmoregulation, regulating the biochemical processes at the cellular level, and activating plant enzymes [17,18,19].

For the present research, 16 elements (Mn, Al, Cu, Zn, Fe, Pb, Cd, As, Cr, Ni, Na, K, Mg, Ca, P, S, Table 2, Table 3 and Table 4) were selected to be determined in the leaves (Table 2) and roots (Table 3) of the lettuce (*Lactuca sativa* L.) treated with antibiotics (ampicillin—AMP; ciprofloxacin—CIP) of different concentrations (AMP I—7.5 mg kg^−1^; AMP II—15 mg kg^−1^; AMP III—30 mg kg^−1^; CIP I—5 mg kg^−1^; CIP II—10 mg kg^−1^; CIP III—20 mg kg^−1^). Also, some macroelements (Mg, Ca, P, and S) were determined in the soil where the plants were grown (Table 4). The results were compared to those of the control plants (grown in the absence of antibiotic treatments). For the soil, the control was a soil in which no antibiotics were added.

In the case of the macroelement content determined in the leaves of the lettuce exposed to antibiotic stress (Table 2), statistical differences were obtained between the control and the treatments and will be further described. The content of K increased significantly for the plants exposed to AMP I and AMP III (over 27% in both cases) in comparison with the control plants. The element K is important for plant survival, both under normal physiological conditions and stress. It is not limited to being an essential element of the plant’s chemical structure. It also plays a vital controlling role in the biochemical and physiological processes that promote plant growth and development [20]. The same antibiotic treatments led to statistically significant increases in the content of P (35.29% for AMP I treatment, 50.47% for AMP III treatment). The element P acts as an activator for over 60 enzymes in plants, helps regulate water content, and minimizes the negative effects of salts on plant health [21]. The content of S increased in the case of the AMP III treatment by 34.15% compared to the control. This element is a fundamental macronutrient for plant growth and metabolism. It assumes a significant role in the production of proteins, enzymes, vitamins, and chlorophyll in plants. As a result, S impacts the plant’s growth, development, nutritional value, and ability to resist or tolerate diseases [22]. The macroelements P and S demonstrate interactions by replacing phospholipids with sulfolipids and galactolipids in cellular membranes during P deficiency stress [23].

The amount of the microelement Mn was reduced in the plants exposed to AMP II (73.79%), CIP I (57.79%), and CIP III (57.82%). The antibiotic CIP III more than doubled the Fe content in lettuce leaves, and AMP I treatment decreased the Fe content by 32.17%, in comparison with the control plants. The CIP I and CIP III treatments induced an extremely high content in the lettuce leaves (over nine times and over three times, respectively). The micronutrients Fe and Zn are essential for the physiological processes of crop plants, though they are needed in very small quantities. The element Fe is crucial for chlorophyll synthesis and the maintenance of chloroplast structure and function. Deficiency in Fe content is a prevalent nutritional issue in many crop plants, contributing to symptoms such as interveinal chlorosis in young leaves, stunted root growth, decreased yield, and lower nutritional quality [22]. The content of the elements As (1.012 mg kg ^−1^) and Ni (20.82 kg ^−1^) increased in the plants exposed to CIP III relative to the control ones. A similar increasing trend, at the same antibiotic treatment, was observed in the content of Cr (5.30 kg ^−1^). Also, the content of Cr was modified but decreased in the case of the treatments with AMP I (38.77%), AMP II (31.33%), CIP I (41.79%), and CIP II (42.19%). The content of Na decreased in the case of CIP treatment at all concentrations (30.4% for CIP I, 28.74% for CIP II, and 68.93% for CIP III). A statistically significant decrease in the content of Na was obtained in the plants treated with AMP II (36.45%).

The elemental analysis in the roots (Table 3) and the soil (Table 4) where the lettuce plants were grown showed variations in their content between treatments and controls. These slight variations in the elemental content were analyzed and demonstrated not to be statistically significantly different from the control (Table 2 and Table 3).

Overall, it was observed that modifications in the elemental content occurred in the leaves of *Lactuca sativa* L. plants treated with the selected antibiotics. The greatest changes were induced by CIP at the highest concentration (CIP III, 20 mg kg^−1^).

Minden et al. demonstrated that even small concentrations of antibiotics in the soil disturb element homeostasis, altering the proportional connections between roots and other plant organs. This disruption may impact metabolic processes and, ultimately, the plant’s performance [1]. Studies on the interactions between various nutrient elements indicate that they influence each other’s uptake, transport, or assimilation. Therefore, it is important to explore the complex interactions between these nutrients to gain a deeper understanding of the sensing and signaling pathways activated in response to changes in nutrient availability [22].

Additionally, the content of both chlorophylls and carotenoids is influenced by the presence and ratio of mineral elements in the plant [24]. Transferring to our study, the changes that occurred in the composition of lettuce leaves during growth, Mn, As, Cr, Na (Table 2), chlorophylls, and total carotenoids (Figure 1) presented a similar pattern. These changes highlight the connection between nutrient availability and plant pigmentation, which can affect plant photosynthesis and overall growth.

### 2.4. Chemometric Evaluation of Antibiotics on the Composition of Lactuca sativa L.

#### 2.4.1. Hierarchical Cluster Analysis (HCA)

**Figure 4:**
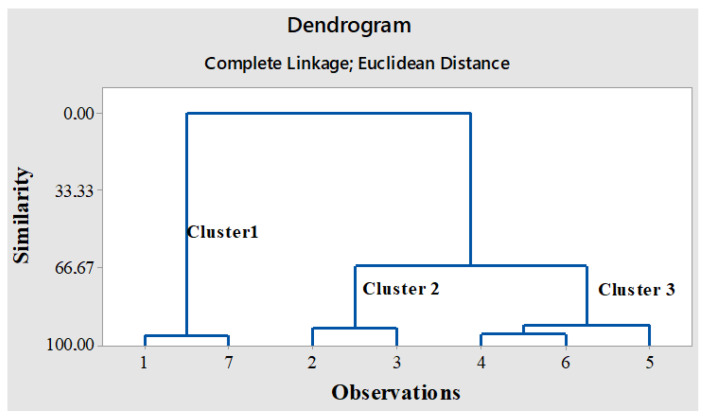
Hierarchical cluster analysis (HCA) of *Lactuca sativa* L. plants treated with different concentrations of ampicillin (AMP) and ciprofloxacin (CIP), in association with bioactive compounds and antioxidant activity. The numbers 1–7 represent the treatments as follows: 1: control plants (untreated); 2: plants grown in soil with AMP I (7.5 mg kg^−1^); 3: plants grown in soil with AMP II (15 mg kg^−1^); 4: plants grown in soil with AMP III (30 mg kg^−1^); 5: plants grown in soil with CIP I (5 mg kg^−1^); 6: plants grown in soil with CIP II (10 mg kg^−1^); and 7: plants grown in soil with CIP III (20 mg kg^−1^).

This analysis builds a hierarchy of data groups with different similarities, usually represented by a dendrogram. The cluster analysis (HCA) (Figure 4) was obtained with a data set for bioactive compounds and antioxidant activity from lettuce plants grown in soil contaminated with AMP or CIP of different concentrations, using Euclidean distance as a measure of similarity. The distance between two clusters was obtained with a complete linkage method.

The HCA dendrogram for the treatments showed that they separated into three distinct clusters. The first cluster included control plants (1) and those grown in soil with CIP III (7); the second cluster included plants grown in soil with AMP I (2) and AMP II (3); and the third cluster included plants grown in soil with AMP III (4), CIP I (5), and CIP II (6). Both clusters 2 and 3 form a superior cluster, in contrast to cluster 1, which is mostly distinct from the other two. The similarity between CIP III and the control sample suggests that while low levels of AMP alter the plant’s metabolism, affecting polyphenols and assimilatory pigments, higher levels enable the plant to cope with stress by upregulating the biosynthesis of new metabolites, which future studies should investigate.

#### 2.4.2. Principal Component Analysis

Principal component analysis (PCA) was conducted using the content of bioactive compounds and antioxidant activity from lettuce plants subjected to abiotic stress induced by AMP and CIP at various concentrations. PCA is a statistical method that diminishes the complexity of the original data set by transforming it into a smaller set of new, orthogonal variables called principal components (PCs). Each PC is constructed from linear combinations of the original variables and is a useful tool to examine relationships between compounds as well as to detect possible outliers [25,26].

**Figure 5:**
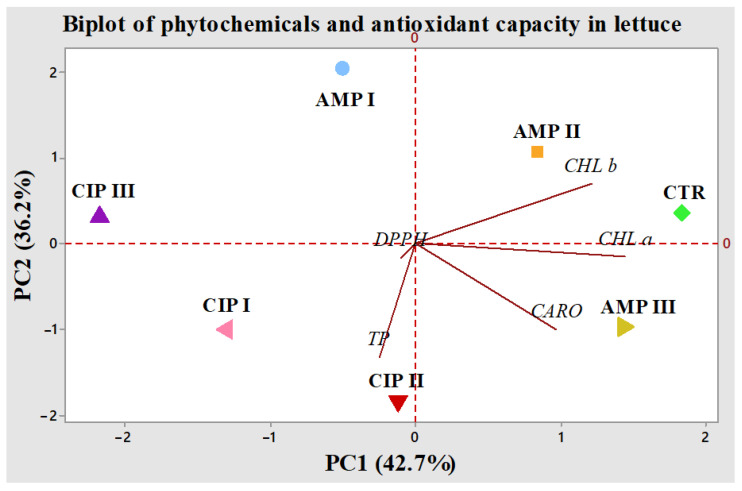
A biplot representation of the bioactive compounds (TP: total polyphenols; *CHL a*: chlorophyll *a*; *CHL b*: chlorophyll *b*; *CARO*: total carotenoids) and antioxidant capacity (DPPH) of *Lactuca sativa* L. (lettuce) treated with antibiotics (ampicillin—AMP; ciprofloxacin—CIP) of different concentrations (AMP I—7.5 mg kg^−1^; AMP II—15 mg kg^−1^; AMP III—30 mg kg^−1^; CIP I—5 mg kg^−1^; CIP II—10 mg kg^−1^; CIP III—20 mg kg^−1^).

The PCA is used to interpret large data sets by reducing the number of variables in the data set to a smaller number of new variables, the so-called principal components (PCs) [27]. The main two principal components are used for graphical interpretation as a PCA biplot that shows the scores and loadings and provides a graphical relationship between the samples and the variables in the data matrix. In the present study, PCA was applied to determine the changes occurring in the composition of lettuce treated with different concentrations of AMP and CIP. A set of five PCs was generated. The PCA biplot of the results is shown in Figure 5.

The first principal component (PC1) exhibited the highest eigenvalue of 2.13, accounting for 42.7% of the total variance, whereas the second principal component (PC2) had an eigenvalue of 1.80, explaining 36.2% of the total variance.

The first component, PC1, was positively correlated with chlorophyll *a* and total carotenoids, while PC2 had a high loading of components from the analyzed variable of total phenolic content and weaker ones from chlorophyll *b* and antioxidant capacity, and it had negative loadings of chlorophyll *a* and total carotenoids.

The direction of the lines means an increase in the concentration of each bioactive compound and the antioxidant capacity. Chlorophyll *b* was clustered in the upper right part of the biplot, and chlorophyll *a* and total carotenoids were clustered in the lower right part. Total polyphenols and antioxidant capacity were located in the lower left quadrant of the biplot. Parameters with vectors pointing in the same direction exhibit a positive correlation with one another.

When it comes to the case analysis after the PCA, it is clear that CIP-treated samples are mostly differentiated by the second component, i.e., assimilatory pigments change, while AMP-treated samples are differentiated by both the first and second components with a higher weight from PC1, i.e., total phenolic content. This finding supports the hypothesis that the two antibiotics lead to totally different changes in the treated plants with different types of coping mechanisms and different impacts on the change in their metabolites.

**Figure 6:**
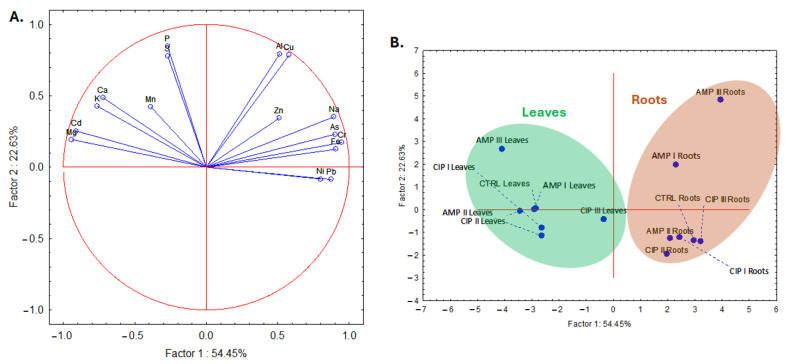
Loading plot (**A**) and case plot (**B**) obtained after the application of PCA to the matrix of the elemental content (Mn, Al, Cu, Zn, Fe, Pb, Cd, As, Cr, Ni, Na, K, Mg, Ca, P, S) determined both in the leaves and roots of the *Lactuca sativa* L. plants treated with antibiotics (ampicillin—AMP; ciprofloxacin—CIP) of different concentrations (AMP I—7.5 mg kg^−1^; AMP II—15 mg kg^−1^; AMP III—30 mg kg^−1^; CIP I—5 mg kg^−1^; CIP II—10 mg kg^−1^; CIP III—20 mg kg^−1^).

To investigate to what extent the antibiotics influence the elemental content in the treated plants, an inductively coupled plasma–optical emission spectrometer (ICP-OES) analysis was conducted, and the results are shown in Table 2 and Table 3. As can be observed from Figure 6, after applying the PCA to the matrix containing the elemental content in both the leaves and roots of the treated plants, a total variance of more than 70% was explained by the first two components: 54% for the first one and about 23% for the second one. Nonmetals such as S and P and to a lesser extent metals such as Al and Cu are explained mostly by the second component and others such as toxic Pb, Cd, As, and Ni but also essential ones such as Mg, K, Ca, Na, Fe, and Cr. Plotting the case scores (Figure 6B) reveals a clear separation of the leaves from the roots, which was expected. However, there is no pattern separation for the two antibiotics, neither for leaves nor for roots, which indicates that in this case, the influence of the two antibiotics on the elemental content was similar. This was different in the case of the polyphenolics (vide supra). Also, in this case, both the first and second components contribute to the spread of the cases in the plot.

## 3. Materials and Methods

### 3.1. Reagents and Materials

Seeds of the *Lactuca sativa* L. variety Attraction (S.C. Agrosem Impex S.R.L., Târgu Mureș, Romania) and commercial garden soil enriched with humus and fertilizer for six weeks (Agro CS, Nové Hony, Slovakia, 50 L) were purchased from the market. The antibiotic standards used for this study were purchased as follows: ampicillin (AMP) was purchased from Antibiotice S.A. (Iași, Romania), and ciprofloxacin (CIP) from Sigma-Aldrich (Hamburg, Germany). To obtain lettuce extracts from plants grown in the presence and absence of antibiotics, ethanol used for the extraction of polyphenols and acetone for the extraction of assimilatory pigments were procured from Chimopar S.A. (Bucharest, Romania). Other chemicals such as Folin–Ciocâlteu reagent, 2,2′-diphenyl-picrylhydrazyl (DPPH), anhydrous sodium carbonate, 6-hydroxy-2, 5, 7, 8-tetramethylchroman-2-carboxylic acid, and gallic acid (GA) were bought from Sigma-Aldrich (Hamburg, Germany) and were used for the characterization of the plant extracts. Methanol was purchased from Chimopar S.A. (Bucharest, Romania). Ultrapure water was prepared using a Direct-Q^®^ 3 UV water purification system (Merck, Darmstadt, Germany).

### 3.2. Conditions for Plant Growth

The test plant was lettuce (*Lactuca sativa* L.). Lettuce seeds were planted in plastic pots loaded with soil (500 g/pot) and contaminated with three different concentrations of ampicillin (7.5 mg kg^−1^—AMP I; 15 mg kg^−1^—AMP II; 30 mg kg^−1^—AMP III) and ciprofloxacin (5 mg kg^−1^—CIP I; 10 mg kg^−1^—CIP II; 20 mg kg^−1^—CIP III). The commercial garden soil used had a pH of 5.5 ± 0.5, the N content was at least 0.1 m/m%, the P_2_O_5_ content was at least 0.01 m/m%, and the K_2_O content was at least 0.03 m/m%. A total of 10 lettuce seeds were sown in each pot. The antibiotics chosen for this study were dissolved in 200 mL of ultrapure water, which was then used to water the pots. Control plants were grown in soil that was not contaminated with antibiotics. Additionally, the plants (both treated and untreated controls) were watered with 100 mL of ultrapure water every 4 days. The antibiotic concentrations used for this research (5–30 mg kg^−1^) were selected to be similar to or lower than those reported in realistic soil samples (0.1–2683 μg kg^−1^ [28]; 2160 μg kg^−1^ [29]). Also, the concentration of drugs (100 mg kg^−1^ paracetamol) with a similar use frequency to antibiotics were taken into account for the assessment of their influence on different crops [30].

The plants in these experiments were grown in a Memmert climate chamber ICH 256 L (Schwabach, Germany) under controlled conditions: a 12/12 h light/dark cycle, 60% humidity, and temperatures of 25 °C during the day and 18 °C at night.

Three replicates of each treatment (treated and control plants) were cultivated. The plant samples were collected 6 weeks after planting, from which the assimilating pigments (chlorophyll *a*, chlorophyll *b*, total carotenoids), the total content of polyphenols, and the antioxidant activity of the polyphenolic extracts were extracted and determined.

### 3.3. Determination of the Bioactive Compounds and Antioxidant Capacity

#### 3.3.1. Measurement of Chlorophyll and Carotenoid Content

The extraction method for chlorophylls (chlorophyll *a* and chlorophyll *b*) and total carotenoids was performed as previously described [31]. The chlorophyll *a*, chlorophyll *b*, and total carotenoid contents in the extracts were quantitatively analyzed using UV-VIS spectroscopy with a UV-VIS T80 spectrophotometer (PG Instruments Limited, Lutterworth, UK). The absorption spectra of the extracts were registered within the wavelength range of 400–750 nm, and the pigment concentrations were determined using the equations established by Lichtenthaler and Buschmann [32].

#### 3.3.2. Obtaining and Evaluating Total Polyphenols

Fresh lettuce leaves (1 g) were minced using liquid nitrogen, and the extraction solvent was added. The resulting mixture was subjected to ultrasound-assisted extraction using an Elma Transsonic T ultrasonic bath (Elma Schmidbauer, Singen, Germany).

The extraction was performed at room temperature for 30 min, using 60% (*v*/*v*) ethanol (15 mL) as an extraction solvent. After extraction, the mixture was centrifuged at 7000 rpm for 10 min, after which the supernatant was decanted and stored in a refrigerator at 4 °C until analysis. All extracts were obtained in triplicate. Following extraction, the mixture was centrifuged at 7000 rpm for 10 min. The supernatant was then decanted and kept in a refrigerator at 4 °C until analysis. All extracts were prepared in triplicate.

The total polyphenol content in the obtained lettuce extracts was measured using the Folin–Ciocalteu method, as outlined by Ivanova et al. [33] and Soran et al. [31].

#### 3.3.3. Assessment of Antioxidant Capacity Using the DPPH Method

The extracted samples were also evaluated for antioxidant capacity using a minorly adjusted version of the method by Brand-Williams et al. [34], as outlined by Soran et al. [31].

### 3.4. Determination of the Elemental Content in Lactuca sativa L. Plants

The samples (leaves, roots, and soil) were first exposed to microwave-assisted acid digestion in a Berghof Speedwave Entry microwave (Berghof Products + Instruments GmbH, Eningen, Germany) equipped with 10 high-pressure TFM™-PTFE-quartz vessels that allowed a maximum pressure and temperature of 40 bar and 230 °C, respectively. The leaf digestion procedure involved adding 0.3 g of each sample into a mixture of 5 mL 69% (*v*/*v*) HNO_3_ (Merck, Darmstadt, Germany) and 3 mL 30% (*v*/*v*) H_2_O_2_. Roots were mineralized by using around 0.05 g of each sample, 5 mL 69% (*v*/*v*) HNO_3_, and 3 mL 30% (*v*/*v*) H_2_O_2_, while soil samples were digested adding around 0.3 g of each sample, 2 mL 37% (*v*/*v*) HCl, 9 mL 69% (*v*/*v*) HNO_3_, 3 mL 40% (*v*/*v*) HF (Merck, Darmstadt, Germany), 1 mL 30% (*v*/*v*) H_2_O_2_, and 2 mL 70% (*v*/*v*) HClO_4_ (Merck, Darmstadt, Germany). Afterward, the microwave protocol followed a four-step heating trend starting from 75 °C to 190 °C. After cooling, the decomposed samples were diluted up to 20–30 mL with ultrapure water (Direct-Q^®^ 3 UV Water Purification System, Merck, Darmstadt, Germany) and further analyzed.

An inductively coupled plasma–optical emission spectrometer PlasmaQuant 9100 Elite High Resolution ICP-OES (Analytik Jena, Jena, Germany) equipped with an Echelle double monochromator, a concentric borosilicate nebulizer, a borosilicate glass cyclonic spray chamber, and a V Shuttle Torch with a 2 mm quartz injector was used to quantify the metal concentrations in the digested samples. The spectrometer was coupled to a Cetac ASX 560 Autosampler (Teledyne CETAC Technologies, Omaha, NE, USA) with 240 positions. The axial configuration was employed to identify the elements at the following spectral lines: Mn II 257.610 nm, Zn II 206.200 nm, Al I 396.152 nm, Cu I 324.754 nm, Fe II 259.940 nm, Pb II 220.353 nm, Cd II 214.441 nm, As I 188.979 nm, Cr II 267.716 nm, Ni II 221.648 nm, Na I 589.592 nm, K I 769.897 nm, Mg I 285.213 nm, Ca II 315.887 nm, P I 213.618 nm, and S I 180.672 nm. Argon was used for plasma generation and as an auxiliary and nebulization gas at a flow rate of 15 L min^−1^, 0.50 L min^−1^, and 0.60 L min^−1^, respectively. Ultrapure water, 1000 mg L^−1^ Multielement standard solution IV (Merck, Darmstadt, Germany), 1000 mg L^−1^ Arsenic standard solution (Merck, Darmstadt, Germany), 10,000 mg L^−1^ Phosphorus standard solution (Merck, Darmstadt, Germany), and 10,000 mg L^−1^ Sulfur standard solution (Merck, Darmstadt, Germany) were used to prepare the stock solutions for calibrations. All measurements were performed in triplicate, and 2 QC standards were selected for the analysis.

### 3.5. Statistical Analysis

The calculations were performed using Microsoft Office Excel 2010 (Microsoft, Redmond, WA, USA). The graphs were drawn in Origin 8 (OriginLab Corporation, Northampton, MA, USA). The values are expressed as the average of three experiments ± standard error (SE). One-way analysis of variance (ANOVA), proceeded by Tukey’s test, was conducted using Minitab 19 (Minitab Ltd., Coventry, UK) to assess statistically significant differences between the obtained averages (*p* < 0.05).

Multivariate analysis was used to gain insight into similarities and differences between treatments. A hierarchical cluster analysis (HCA) was first performed, followed by a principal component analysis (PCA). HCA and PCA analyses were both carried out using Minitab 19. All statistical tests were considered significant at *p* < 0.05.

## 4. Conclusions

This research evaluated the variation in the amount of bioactive compounds, antioxidant activity, and elemental content in lettuce (*Lactuca sativa* L.) plants grown in soil contaminated with different concentrations of ampicillin and ciprofloxacin, compared to control plants. The results demonstrate that plants grown in antibiotic-contaminated soil showed a significant decrease in chlorophyll content compared to control plants. These findings suggest that the plants showed stress due to the presence of antibiotics, impacting their photosynthetic efficiency. A significant 26.73% reduction in total carotenoids was observed only in plants treated with ampicillin at 7.5 mg kg^−1^. This suggests that the presence of antibiotics can impair carotenoid synthesis, which is particularly concerning under stress conditions, as carotenoids are key in protecting plants from oxidative damage and photoinhibition.

At low concentrations of ampicillin (7.5 mg kg^−1^), polyphenol biosynthesis is inhibited, while at higher concentrations, plants respond to stress by upregulating polyphenol production. For ciprofloxacin, polyphenol upregulation occurred at all tested concentrations, suggesting a consistent stress response across the different antibiotic levels. These findings highlight the dynamic role of polyphenols in plant stress adaptation, particularly in response to antibiotic contamination.

This study demonstrated that even low concentrations of antibiotics in soil significantly altered the elemental content in lettuce leaves. The most significant changes occurred in plants treated with ciprofloxacin at the highest concentration (20 mg kg^−1^). These alterations affected both macro- and microelements, influencing plant growth and metabolism. Additionally, the study highlights the importance of exploring the interactions between multiple nutrient elements and bioactive compounds and their important role in plant sensing. Understanding these interactions is important for assessing the broader implications of antibiotic contamination in agricultural systems and its potential impact on food quality and ecosystem health. In conclusion, bioactive compound content and antioxidant capacity depend on both the type and concentration of antibiotics in the soil.

## Figures and Tables

**Table 1:** Correlation of assimilatory pigments (chlorophyll *a*, chlorophyll *b*, total carotenoids), total polyphenols, and antioxidant capacity determined in the *Lactuca sativa* L. plants treated with ampicillin (AMP) and ciprofloxacin (CIP) of different concentrations (I, II, III) with those determined in control plants, untreated with the selected antibiotics.

Treatment	Chlorophyll *a*	Chlorophyll *b*	Total Carotenoids	Total Polyphenols	Antioxidant Capacity
**AMP I**	−0.25965	−0.33254	−0.26195	−0.41667	−0.83772
**AMP II**	−0.12963	−0.10701	−0.11536	−0.05165	−0.77261
**AMP III**	−0.02181	−0.24213	0.05312	0.67884	−0.58888
**CIP I**	−0.30192	−0.85292	−0.09598	0.74119	−0.62583
**CIP II**	−0.18758	−0.69982	0.04893	0.96855	−0.55188
**CIP III**	−0.47429	−0.97569	−0.24118	0.37449	0.0113

Treatments with antibiotics (ampicillin—AMP; ciprofloxacin—CIP) of different concentrations: AMP I—7.5 mg kg^−1^; AMP II—15 mg kg^−1^; AMP III—30 mg kg^−1^; CIP I—5 mg kg^−1^; CIP II—10 mg kg^−1^; CIP III—20 mg kg^−1^.

**Table 2:** Elemental (Mn, Al, Cu, Zn, Fe, Pb, Cd, As, Cr, Ni, Na, K, Mg, Ca, P, S) content (mg kg^−1^) in *Lactuca sativa* L. leaves grown in the presence of antibiotics.

Elemental Content (mg kg^−1^) ± SE	Treatment Applied						
	Control	AMP I	AMP II	AMP III	CIP I	CIP II	CIP III
**Mn**	140.43 ^a^ ± 7.71	90.29 ^ab^ ± 1.37	36.78 ^c^ ± 15.73	105.79 ^ab^ ± 14.65	59.28 ^bc^ ± 1.58	140.24 ^a^ ± 15.30	59.23 ^bc^ ± 5.11
**Al**	2107.88 ^a^ ± 1163.25	318.99 ^a^ ± 128.15	110.43 ^a^ ± 17.34	1252.88 ^a^ ± 598.30	527.26 ^a^ ± 191.48	109.03 ^a^ ± 27.97	102.57 ^a^ ± 9.36
**Cu**	13.38 ^ab^ ± 0.39	6.01 ^ab^ ± 1.34	3.26 ^b^ ± 0.25	21.69 ^a^ ± 8.41	5.07 ^b^ ± 1.52	3.89 ^b^ ± 0.83	2.67 ^b^ ± 0.55
**Zn**	128.26 ^a^ ± 15.48	130.41 ^a^ ± 7.24	102.01 ^a^ ± 0.03	138.56 ^a^ ± 13.87	101.10 ^a^ ± 10.06	101.13 ^a^ ± 2.41	98.31 ^a^ ± 2.46
**Fe**	163.01 ^b^ ± 22.76	110.56 ^c^ ± 4.72	114.71 ^bc^ ± 4.87	162.61 ^bc^ ± 10.78	125.06 ^bc^ ± 10.22	135.06 ^bc^ ± 0.75	546.32 ^a^ ± 4.12
**Pb**	0.54 ^c^ ± 0.06	0.70 ^bc^ ± 0.07	1.30 ^bc^ ± 0.07	0.63 ^bc^ ± 0.12	2.33 ^b^ ± 0.80	1.23 ^bc^ ± 0.51	5.46 ^a^ ± 0.07
**Cd**	1.01 ^ab^ ± 0.05	0.93 ^ab^ ± 0.03	0.812 ^ab^ ± 0.02	1.20 ^a^ ± 0.14	0.91 ^ab^ ± 0.01	0.99 ^ab^ ± 0.01	0.73 ^b^ ± 0.16
**As**	0.44 ^bc^ ± 0.01	0.44 ^bc^ ± 0.005	0.33 ^c^ ± 0.02	0.57 ^b^ ± 0.07	0.38 ^c^ ± 0.01	0.43 ^bc^ ± 0.01	1.01 ^a^ ± 0.01
**Cr**	1.44 ^b^ ± 0.11	0.88 ^cd^ ± 0.03	0.99 ^cd^ ± 0.09	1.28 ^bc^ ± 0.14	0.84 ^cd^ ± 0.03	0.83 ^d^ ± 0.01	5.30 ^a^ ± 0.13
**Ni**	1.07 ^b^ ± 0.01	0.66 ^b^ ± 0.001	1.17 ^b^ ± 0.10	0.96 ^b^ ± 0.13	0.60 ^b^ ±0.04	0.56 ^b^ ± 0.01	20.82 ^a^ ± 0.60
**Na**	5597.06 ^b^ ± 202.64	4779.76 ^bc^ ± 186.92	3325.60 ^d^ ± 225.70	5565.07 ^b^ ± 327.92	3643.17 ^cd^ ± 132.44	3729.63 ^cd^ ± 390.51	8841.50 ^a^ ± 118.74
**K**	142,885.34 ^b^ ± 3944.14	182,686.34 ^a^ ± 5816.71	117,905.34 ^b^ ± 261.03	182,045.24 ^a^ ± 14,100.03	125,965.59 ^b^ ± 8809.50	129,186.64 ^b^ ± 10365.38	125,904.20 ^b^ ± 2159.47
**Mg**	5720.66 ^ab^ ± 138.56	5172.65 ^b^ ± 268.87	4708.73 ^b^ ± 72.10	7028.67 ^a^ ± 899.51	4961.66 ^b^ ± 28.98	5266.66 ^ab^ ± 27.69	4094.16 ^b^ ± 4.91
**Ca**	20,958.74 ^a^ ± 1719.81	18,946.72 ^a^ ± 1429.18	23,740.70 ^a^ ± 2642.03	25,886.70 ^a^ ± 3334.29	21,266.70 ^a^ ± 2938.78	23,966.70 ^a^ ± 2026.29	20,556.31 ^a^ ± 212.96
**P**	6018.00 ^c^ ± 28.11287	8142.00 ^ab^ ± 253.28	6159.50 ^c^ ± 6.64	9055.00 ^a^ ± 863.50	7009.50 ^bc^ ± 34.34	6604.00 ^bc^ ± 142.97	7426.66 ^abc^ ± 162.91
**S**	2978.3 ^b^ ± 100.17	2917.8 ^b^ ± 61.52	3146.30 ^b^ ± 27.07	3995.30 ^a^ ± 336.77	3161.30 ^b^ ± 21.36	3215.30 ^b^ ± 79.48	3275.30 ^b^ ± 50.96

Treatments with antibiotics (ampicillin—AMP; ciprofloxacin—CIP) of different concentrations: AMP I—7.5 mg kg^−1^; AMP II—15 mg kg^−1^; AMP III—30 mg kg^−1^; CIP I—5 mg kg^−1^; CIP II—10 mg kg^−1^; CIP III—20 mg kg^−1^. The results represent the average of three independent replicate experiments ± standard error (SE). The letters (a, b, c, and d) next to the means indicate grouping information using the Tukey method, and means that do not have a common letter are significantly different.

**Table 3:** Elemental (Mn, Al, Cu, Zn, Fe, Pb, Cd, As, Cr, Ni, Na, K, Mg, Ca, P, S) content (mg kg^−1^ FW) in *Lactuca sativa* L. roots grown in the presence of antibiotics.

Elemental Content (mg kg^−1^ FW) ± SE	Treatment Applied						
	Control	AMP I	AMP II	AMP III	CIP I	CIP II	CIP III
**Mn**	78.97 ^a^ ± 18.90	75.87 ^a^ ± 26.72	37.52 ^a^ ± 6.91	101.44 ^a^ ± 11.39	62.87 ^a^ ± 26.10	68.04 ^a^ ± 17.46	79.92 ^a^ ± 20.61
**Al**	1547.37 ^a^ ± 439.68	5764.38 ^a^ ± 4079.54	899.56 ^a^ ± 257.93	12998.65 ^a^ ± 11325.61	1605.50 ^a^ ± 575.59	1244.50 ^a^ ± 544.21	1402.35 ^a^ ± 361.92
**Cu**	16.19 ^a^ ± 1.10	77.89 ^a^ ± 47.10	20.08 ^a^ ± 4.08	131.99 ^a^ ± 112.71	26.68 ^a^ ± 7.35	18.18 ^a^ ± 0.33	25.05 ^a^ ± 6.76
**Zn**	172.14 ^a^ ± 14.96	160.31 ^a^ ± 19.33	138.09 ^a^ ± 18.59	148.73 ^a^ ± 49.58	139.01 ^a^ ± 35.92	129.09 ^a^ ± 22.72	107.13 ^a^ ± 9.89
**Fe**	1003.93 ^a^ ± 251.94	893.97 ^a^ ± 243.30	481.91 ^a^ ± 153.54	1254.12 ^a^ ± 200.17	1415.02 ^a^ ± 898.05	619.92 ^a^ ± 197.01	1060.94 ^a^ ± 262.39
**Pb**	5.59 ^a^ ± 2.35	4.21 ^a^ ± 0.66	11.06 ^a^ ± 7.88	9.30 ^a^ ± 5.57	6.35 ^a^ ± 2.58	8.70 ^a^ ± 6.09	7.85 ^a^ ± 3.87
**Cd**	0.37 ^a^ ± 0.04	0.67 ^a^ ± 0.02	0.73 ^a^ ± 0.23	0.55 ^a^ ± 0.17	0.62 ^a^ ± 0.13	0.53 ^a^ ± 0.19	0.41 ^a^ ± 0.04
**As**	2.13 ^a^ ± 0.46	2.50 ^a^ ± 0.31	1.02 ^a^ ± 0.25	2.14 ^a^ ± 0.23	1.57 ^a^ ± 0.50	1.25 ^a^ ± 0.26	2.01 ^a^ ± 0.46
**Cr**	14.35 ^a^ ± 1.15	10.95 ^a^ ± 2.42	15.92 ^a^ ± 3.57	22.08 ^a^ ± 7.05	10.72 ^a^ ± 0.71	9.96 ^a^ ± 0.79	17.52 ^a^ ± 4.74
**Ni**	10.94 ^a^ ± 0.46	15.82 ^a^ ± 7.32	18.11 ^a^ ± 3.02	14.12 ^a^ ± 4.51	13.92 ^a^ ± 3.24	8.47 ^a^ ± 1.05	28.26 ^a^ ± 15.17
**Na**	12,912.58 ^a^ ± 398.59	10,126.62 ^a^ ± 1511.03	10,568.05 ^a^ ± 1207.88	20,530.33 ^a^ ± 8280.66	10,174.92 ^a^ ± 555.81	10,913.44 ^a^ ± 1085.41	12,559.47 ^a^ ± 3552.92
**K**	53,530.93 ^a^ ± 9493.49	64,769.80 ^a^ ± 14727.86	79,738.69 ^a^ ± 2485.69	133,132.41 ^a^ ± 29853.89	80,383.12 ^a^ ± 20190.21	81,342.37 ^a^ ± 22566.38	85,063.88 ^a^ ± 21431.83
**Mg**	3142.59 ^a^ ± 507.63	2820.96 ^a^ ± 370.20	2055.60 ^a^ ± 319.18	2066.94 ^a^ ± 130.65	2269.33 ^a^ ± 696.52	1687.07 ^a^ ± 226.85	1698.66 ^a^ ± 191.82
**Ca**	17,184.07 ^a^ ± 1634.82	17,701.92 ^a^ ± 2967.58	16,506.80 ^a^ ± 3385.47	21,579.81 ^a^ ± 5342.96	14,378.03 ^a^ ± 2579.51	14,366.89 ^a^ ± 863.74	17,821.67 ^a^ ± 1519.71
**P**	4685.18 ^a^ ± 574.79	7642.63 ^a^ ± 398.54	6033.33 ^a^ ± 163.47	9087.04 ^a^ ± 2484.91	5714.54 ^a^ ± 987.05	5171.82 ^a^ ± 1221.39	6203.47 ^a^ ± 717.95
**S**	2751.48 ^a^ ± 19.15	3797.57 ^a^ ± 309.67	3115.33 ^a^ ± 316.63	3544.99 ^a^ ± 956.64	2913.82 ^a^ ± 613.82	2744.33 ^a^ ± 594.06	2487.65 ^a^ ± 194.93

Treatments with antibiotics (ampicillin—AMP; ciprofloxacin—CIP) of different concentrations: AMP I—7.5 mg kg^−1^; AMP II—15 mg kg^−1^; AMP III—30 mg kg^−1^; CIP I—5 mg kg^−1^; CIP II—10 mg kg^−1^; CIP III—20 mg kg^−1^. The results represent the average of three independent replicate experiments ± standard error (SE). The letter next to the average stands for statistical differences, as in Table 2.

**Table 4:** Elemental (Mg, Ca, P, S) content (mg kg^−1^ soil) in the soil where the plants *Lactuca sativa* L. were grown in the presence of antibiotics.

Elemental Content (mg kg^−1^ Soil) ± SE	Treatment Applied						
	Control	AMP I	AMP II	AMP III	CIP I	CIP II	CIP III
**Mg**	1214.59 ^a^ ± 264.55	971.29 ^a^ ± 203.67	846.94 ^a^ ± 201.77	1455.62 ^a^ ± 330.53	1643.99 ^a^ ± 448.53	1185.34 ^a^ ± 274.10	1178.17 ^a^ ± 377.37
**Ca**	23,555.90 ^a^ ± 6614.20	17,047.91 ^a^ ± 2587.79	18,361.22 ^a^ ± 3970.23	27,037.06 ^a^ ± 6555.50	31,227.57 ^a^ ± 2624.23	22,640.52 ^a^ ± 2884.21	21,594.44 ^a^ ± 6270.07
**P**	607.08 ^a^ ± 93.20	600.67 ^a^ ± 140.70	421.09 ^a^ ± 76.29	969.31 ^a^ ± 99.83	816.68 ^a^ ± 68.70	705.67 ^a^ ± 146.46	653.70 ^a^ ± 94.95
**S**	4083.82 ^a^ ± 942.25	3263.23 ^a^ ± 429.96	3563.77 ^a^ ± 279.95	4481.38 ^a^ ± 1026.65	3766.59 ^a^ ± 482.68	3699.08 ^a^ ± 321.25	3391.38 ^a^ ± 215.02

Soil where *Lactuca sativa* L. plants treated with antibiotics (ampicillin—AMP; ciprofloxacin—CIP) of different concentrations were grown: AMP I—7.5 mg kg^−1^; AMP II—15 mg kg^−1^; AMP III—30 mg kg^−1^; CIP I—5 mg kg^−1^; CIP II—10 mg kg^−1^; CIP III—20 mg kg^−1^. The results represent the average of three independent replicate experiments ± standard error (SE). The letter next to the average stands for statistical differences, as in Table 2.

## Data Availability

All data are included in the article.
